# Impact of brain arousal and time-on-task on autonomic nervous system activity in the wake-sleep transition

**DOI:** 10.1186/s12868-018-0419-y

**Published:** 2018-04-11

**Authors:** Jue Huang, Christine Ulke, Christian Sander, Philippe Jawinski, Janek Spada, Ulrich Hegerl, Tilman Hensch

**Affiliations:** 1 Department of Psychiatry and Psychotherapy, University of Leipzig, Semmelweisstrasse 10, 04103 Leipzig, Germany; 2 Depression Research Centre, German Depression Foundation, Leipzig, Germany

**Keywords:** VIGALL 2.1, Brain arousal, EEG-vigilance stages, Time-on-task effect, Heart rate, Skin conductance level

## Abstract

**Background:**

Autonomic nervous system (ANS) activity has been shown to vary with the state of brain arousal. In a previous study, this association of ANS activity with distinct states of brain arousal was demonstrated using 15-min EEG data, but without directly controlling for possible time-on-task effects. In the current study we examine ANS-activity in fine-graded EEG-vigilance stages (indicating states of brain arousal) during two conditions of a 2-h oddball task while controlling for time-on-task. In addition, we analyze the effect of time-on-task on ANS-activity while holding the level of brain arousal constant.

**Methods:**

Heart rate and skin conductance level of healthy participants were recorded during a 2-h EEG with eyes closed under simultaneous presentation of stimuli in an ignored (N = 39) and attended (N = 39) oddball condition. EEG-vigilance stages were classified using the Vigilance Algorithm Leipzig (VIGALL 2.1). The time-on-task effect was tested by dividing the EEG into four 30-min consecutive time blocks. ANS-activity was compared between EEG-vigilance stages across the entire 2 h and within each time block.

**Results:**

We found a coherent decline of ANS-activity with declining brain arousal states, over the 2-h recording and in most cases within each 30-min block in both conditions. Furthermore, we found a significant time-on-task effect on heart rate, even when arousal was kept constant. It was most pronounced between the first and all subsequent blocks and could have been a consequence of postural change at the beginning of the experiment.

**Conclusion:**

Our findings contribute to the validation of VIGALL 2.1 using ANS parameters in 2-h EEG recording under oddball conditions.

**Electronic supplementary material:**

The online version of this article (10.1186/s12868-018-0419-y) contains supplementary material, which is available to authorized users.

## Background

Brain arousal fundamentally affects all dimensions of human behavior [1]. It can best be assessed using electroencephalography (EEG). According to the scoring systems by Rechtschaffen and Kales [2] and the American Academy of Sleep Medicine [3], different sleep stages can be differentiated, but only one uniform wake state has been described. However, it has been demonstrated that different states of arousal can be discerned during the waking state [4–7]. For example, the wake-sleep transition is a period which is characterized by small arousal fluctuations [8] before stable sleep begins. To date, only a few studies have examined states of arousal in the period before sleep onset, which could be due to a lack of reliable research methods.

To fill this gap, the Vigilance Algorithm Leipzig (VIGALL), an EEG- and electrooculogram (EOG)-based algorithm, was introduced by Hegerl and colleagues [9–13]. VIGALL allows the automatic classification of brain arousal (assessed as EEG-vigilance stages) in the wake-sleep transition (see Table 1). Markers of autonomic nervous system (ANS) arousal (heart rate: HR; skin conductance levels: SCL; and temperature) can simultaneously be assessed.

**Table 1:** Assessment of brain arousal states by applying VIGALL 2.1

EEG-vigilance stage	Corresponding behavioral state	EEG-characteristics
0	Cognitively active wakefulness	Low amplitude, desynchronized non-alpha EEG without horizontal SEM
A1	Relaxed wakefulness	Occipital dominant alpha activity
A2		Shifts of alpha to central and frontal cortical areas
A3		Continued frontalization of alpha
B1	Drowsiness	Low amplitude, desynchronized non-alpha EEG with SEM
B2/3		Dominant delta- and theta-power
C	Sleep onset	Occurrence of K-complexes and sleep spindles

*VIGALL* Vigilance Algorithm Leipzig, *EEG* electroencephalogram, *SEM* slow eye movements

Although situational factors substantially contribute to arousal states, the trait aspect of arousal is well-known and, in fact, a genetic component has been suggested [1, 14]. Moreover, arousal and regulatory systems have been posited as a fundamental domain for classifying mental disorders by the Research Domain Criteria Project (RDoC) of the National Institute of Mental Health (NIMH) [15]. In line with this, the Arousal Regulation Model posits a pathophysiological role of brain arousal in affective disorders and attention-deficit/hyperactivity disorder (ADHD) [9, 12, 16, 17]. VIGALL has been utilized in several clinical studies to identify distinct patterns of brain arousal regulation across these psychiatric disorders. For example, an unstable regulation of brain arousal was found in patients with bipolar disorder during manic episodes [18] and in patients with ADHD [19]. In contrast, a hyperstable pattern of arousal regulation was observed in patients with depression [20, 21] and bipolar patients during depressive episodes [18, 22].

Earlier versions of VIGALL have been validated using EEG-fMRI data [10], in a PET study [23], against evoked potentials [24] and relating ANS parameters to different EEG-vigilance stages [11].

Olbrich et al. [11] compared HR and SCL of healthy individuals in different EEG-vigilance stages using an earlier version of VIGALL to 15-min resting EEG data. High EEG-vigilance stages showed significantly higher HR and SCL activity in comparison to lower EEG-vigilance stages in regression analyses, but this was not the case for the comparisons between stage 0 and A1 (possibly due to the misclassification of stage 0, according to the authors). Concerning SCL, however, the effect of time explained more variance than did EEG-vigilance stages. Additionally, a direct effect of time on both central [25] and ANS arousal [26, 27] is well established elsewhere. However, no study to date has directly assessed this possible effect on ANS activity independent of the co-occurring arousal decline.

To gain more insight into the direct effect of time (i.e. time-on-task) on ANS activity, we analyzed a previously published dataset [24] of 2-h EEG data recorded in two conditions of an oddball experiment while controlling for arousal. We divided the 2-h EEG recording into four consecutive 30-min blocks, which enabled us to examine the time-on-task effect. The analyses were restricted to particular EEG-vigilance stages in order to control for the additional influence of brain arousal on ANS activity. The extended recording period also ensured a reliable comparison of ANS activity between different EEG-vigilance stages, since enough data from each arousal state could be obtained. Specifically, we analyzed whether the findings by Olbrich, (i.e. the decrease in ANS activity with the decline of EEG-vigilance stages) can be replicated in each of the four time blocks.

We hypothesize that there will be a decrease in ANS activity with the decline of EEG-vigilance stages over 2 h as well as within each time block (Hypothesis 1). We further hypothesize that there is a significant time-on-task effect on ANS activity across the four 30-min blocks when restricting the analysis to individual EEG-vigilance stages (Hypothesis 2). We are particularly interested in differences in ANS activity between EEG-vigilance stages 0 and A1 on account of the recently released VIGALL 2.1 which has greater classification accuracy. To this end, we set out to reexamine the shifts in ANS activity between fine-graded EEG-vigilance stages, for the first time in two different conditions (ignored and attended) of an oddball task.

## Methods

### Subjects

Healthy volunteers were recruited via local and online advertisements. None of the subjects reported a history of sleep disorder, psychiatric or neurological diseases, or current intake of psychotropic medication. All subjects were required to participate in two EEG recordings (one ignored and one attended oddball condition, see below) with an interval of 7 days between the recordings. These two recordings were performed in a pseudorandom order. Not all subjects participated in the second session due to lack of compliance or availability, leaving 45 subjects in the ignored and 49 subjects in the attended condition. Within these participants, those who exhibited insufficient arousal variability during the 2-h recording (i.e. too much EEG-vigilance stage A1; n = 6 in the ignored and n = 10 in the attended condition) were further excluded. As result, the final sample consisted of 39 subjects in the ignored (22 females, age = 23.90 ± 3.93) and 39 in the attended condition (24 females, age = 24.46 ± 4.44), respectively. The study was approved by the local ethics committee of the University of Leipzig (075-13-11032013). Each subject gave written informed consent prior to the first recording. All subjects received 20€ or course credits (psychology students) for their participation.

### Procedure

The 2-h EEG recordings began between 1 and 4 p.m. in a light-dimmed and sound attenuated room. The temperature in the booth was maintained around 25 °C at the beginning of each recording. For each individual, the time of assessment was the same in both sessions. During the EEG recording, subjects lay comfortably on a lounge chair while standard (500 Hz) and deviant (1000 Hz) tone were presented in an oddball sequence with stimuli probabilities of 80 and 20% respectively. In the ignored condition, subjects were instructed to ignore the tones, while in the attended condition they performed a simple cognitive task such as pressing a button to target stimuli. At the beginning of each recording, the body position was changed from upright to laid-back. During the recording, subjects were instructed to close their eyes, relax and not fight against an urge to sleep. When subjects did fall asleep, they were woken up after 5 min and asked to answer a common question (e.g. today’s date) before they were allowed to continue the task. This process was repeated until the end of the experiment in order to acquire enough data from each arousal state.

### EEG-recording and EEG-vigilance staging

The EEG was recorded at 1000 Hz with Ag/AgCl electrodes and DC amplifiers (QuickAmp; Brain Products GmbH, Gilching, Germany) from 31 sites (Fp1, Fp2, F3, F4, F7, F8, Fz, FC1, FC2, FC5, FC6, C3, C4, T7, T8, Cz, FT9, FT10, CP5, CP6, TP9, TP10, P3, P4, P7, P8, Pz, O1, O2, PO9, PO10) according to the extended international 10–20 system using EasyCap (EASYCAP Brain Products GmbH, Gilching, Germany), and referenced against common average. Impedance of each electrode was kept below 10 kΩ. Bipolar electrodes were placed laterally to the left and right eyes to monitor horizontal eye movements and above and below the right eye to monitor vertical eye movements.

EEG data were analyzed using BrainVision Analyzer 2.1 software (Brain Products GmbH, Gilching, Germany). First, the EEG raw data were pre-processed according to standard operating procedures (see VIGALL manual [7] or refer to Additional file 1). After that, all 1-s EEG-segments were classified into seven different EEG-vigilance stages using VIGALL 2.1 (available at http://research.uni-leipzig.de/vigall/).

### ANS parameters

To assess the R–R intervals of HR (in ms), an electrocardiogram (ECG) was recorded at a 1000 Hz sampling rate using a bipolar channel of the QuickAmp amplifier. Electrodes were placed on both forearms. R-peaks were marked using the CB correction module of BrainVision Analyzer (Brain Products GmbH, Gilching, Germany). The results were visually checked and corrected if necessary.

To assess SCL (in µSiemens), a bipolar channel of the QuickAmp amplifier was used with a constant voltage of 0.5 V (GSR module, Brain Products GmbH, Gilching, Germany). Two Ag/AgCl electrodes (with an overall diameter of 13 mm) were placed at the thenar and hypothenar of the non-dominant hand. A low pass filter of 1 Hz was applied to exclude phasic components of the electrodermal activity due to stimuli presentation (in both conditions) and response (only in the attended condition).

Segments identified as artifacts in the EEG were also marked as artifacts in the ECG and SCL channels. Only artifact-free segments were used in further analyses. VIGALL also provides calculations for R–R intervals and SCL values: R–R interval was computed as the mean of the R–R intervals across three consecutive artifact-free 1-s segments. The HR was calculated for each segment (indexed by 60,000/R–R intervals in ms). SCL value was computed as mean of all data points in each 1-s segment.

In order to account for the considerable degree of variability in SCL raw values between subjects, for each subject SCL values were z-transformed against the mean and standard deviation over 2 h when assessing overall SCL differences between EEG-vigilance stages. SCL values were also z-transformed against the mean and standard deviation in each corresponding time block for each subject when differences within each time block were examined.

### Statistical analysis

A minimum criterion of 10 epochs for each EEG-vigilance stage was set in order to obtain reliable HR and SCL values. Subjects with an insufficient number of epochs were excluded from the comparisons of respective stages. This step resulted in different sample sizes for each EEG-vigilance stage. Some stages, such as A1 and B1, which were frequent, contained more subjects, whereas others, especially A3 and C, which rarely occurred, had fewer subjects (see Additional file 2).

To analyze differences in ANS activity between EEG-vigilance stages across the entire 2 h and within each time block, paired sample (within subject) *t* tests were used. The different sample sizes in the EEG-vigilance stages precluded adequate stage comparisons (due to listwise deletion) by repeated measures analyses of variance (rmANOVAs). Hence, comparisons were only made for pairs with a sufficient sample size (n > 10). All statistical analyses were conducted using IBM SPSS Statistics version 20 (IBM, Armonk, NY, USA).

The time-on-task effect on HR and SCL was analyzed with rmANOVAs. In these analyses, the arousal stage was kept constant by restricting the analyses to a respective EEG-vigilance stage across the four consecutive time blocks (min 1–30, min 31–60, min 61–90, min 91–120). However, these analyses could only be performed in the EEG-vigilance stages A1, A2, B1 and B2/3 in the ignored condition and in stages A1, B1 and B2/3 in the attended condition because the sample sizes (n ≤ 10) across all four blocks were insufficient in the remaining stages. When significant main effects were present, post hoc tests for multiple comparisons were conducted with adjustments for significance level using the Bonferroni method (*p *< 0.0125). When analyzing the time-on-task effect on SCL we did not z-transform the data for two reasons. First, the time course and percentage of low EEG-vigilance stages in each time block may have increased with time (see Additional file 2), possibly resulting in a smaller z-score in low stages in earlier versus later time blocks. This could have led to an artificial time effect. Second, because we used rmANOVAs to examine within-subject effects over time, the inter-individual variations likely had little influence on the results.

## Results

### HR and SCL between EEG-vigilance stages during the total 2-h EEG recording

**Fig. 1:**
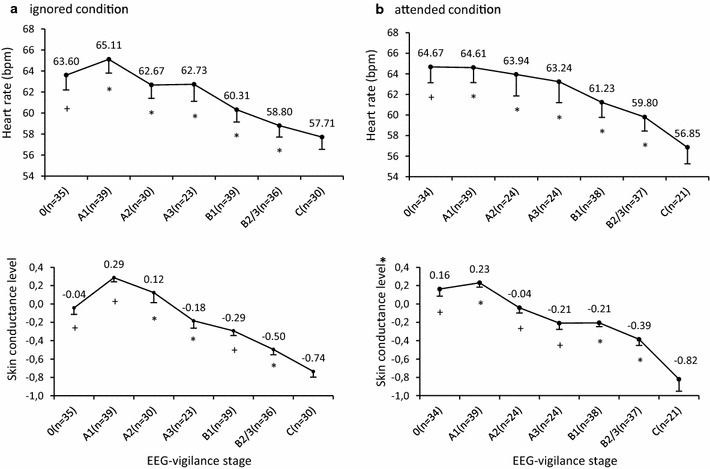
Averaged HR and SCL over 2-h in EEG-vigilance stage. The line diagrams show the mean of heart rate (HR) and skin conductance level (SCL, z transformed) in corresponding EEG-vigilance stages in ignored (**a**) and attended (**b**) condition. To note, the values were calculated based on different subjects. The numbers of subjects in each stage are shown in parentheses. Error bars reflect standard error of the means. The asterisk and plus sign represent results of pared sample *t* tests, which asterisk means significant different between this stage and all other lower stages, while plus sign means some of the comparisons between this stage and other lower stages reached significance level. Each pared sample *t* test was only made for pairs with a sufficient sample size (n > 10), therefore the degree of freedoms were different for each pair. For more details please refer to the Table S2 (ignored condition) and Table S3 (attended condition) in Additional file 3

*In the ignored condition*, the analyses of HR across EEG-vigilance stages revealed a continuous decline from high (A1) to low (C) EEG-vigilance stages, with 14 out of 15 pair-wise comparisons (except pair B2/3 vs. C, *p *= .103) reaching the significance level (1.49E−13 ≤ *p* ≤ .023). HR in stage 0 was significantly higher than in the other far lower stages, i.e. B1, B2/3 and C (8.91E−8 ≤ *p *≤ 8.05E−7), whereas no significant differences were found in comparisons with neighboring lower stages, i.e. A1, A2 and A3 (.063 ≤ *p* ≤ .260). Additionally, the HR value in stage 0 was even lower than in A1 and A2. For SCL, we also observed a continuous decrease from stage A1 to C (1.91E−13 ≤ *p* ≤ .019) with the exception of comparisons between some neighboring stages, i.e. A1 versus A2 (*p *= .134), A3 versus B1 (*p *= .156). SCL in stage 0 was higher than in other stages (except stage A1), wherein the comparisons with stage B1, B2/3 and C reached significance level (1.62E−6 ≤ *p *≤ .005). SCL increased significantly from stage 0 to A1 (*p *= 3.12E−5). A visual illustration of ANS activity in the ignored condition is shown in Fig. 1. The detailed results of paired *t* tests are summarized in Table S2 in Additional file 3.

*In the attended condition*, a significant continuous decrease in HR from stage A1 to C was obtained from all pair-wise comparisons (6.80E−11 ≤ *p* ≤ .021). A significantly higher HR in stage 0 relative to stages ranging from A2 to C was observed (3.64E−8 ≤ *p *≤ 1.25E−6), except for the comparison of 0 versus A2 (*p *= .564). A slightly higher but non-significant HR in stage A1 than in stage 0 was seen (*p *= .301). Similar results regarding SCL were demonstrated in 12 out of 15 pair-wise comparisons of stages ranging between A1 and C (2.86E−7 ≤ *p *≤ .035). Exceptions included some comparisons between neighboring stages, i.e. A2 versus B1 (*p *= .068), A3 versus B1 (*p *= .607), A3 versus B2/3 (*p *= .165). A significant higher SCL in stage 0 was obtained as compared to stages from A2 to C (4.82E−5 ≤ *p *≤ .002), with exception of stage A2 (*p *= .106). A non-significant increasing trend in SCL from stage 0 to A1 was observed (*p *= .288). A graphical representation is shown in Fig. 1. The detailed results of paired *t* tests are provided in Table S3 in Additional file 3.

### The time-on-task effect on HR and SCL: restricting analyses to certain EEG-vigilance stages

**Fig. 2:**
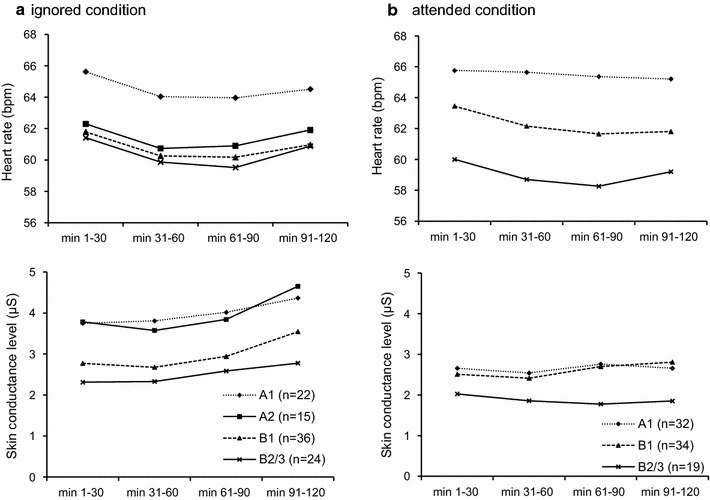
Averaged HR and SCL within 30-min time block in EEG-vigilance stage. Heart rate (HR) and skin conductance level (SCL) values were calculated over available subjects in each time block (min 1–30, min 31–60, min 61–90, min 91–120) during corresponding EEG-vigilance stages in ignored (**a**) and attended (**b**) condition. The numbers of available subjects are presented in parentheses. To note, the tests in other stages could not be conducted due to insufficient sample size (n ≤ 10)

Results of rmANOVAs for assessing the *time*-*on*-*task* effect are summarized in Table 2. HR and SCL in four time blocks in corresponding EEG-vigilance stages are presented in Fig. 2. Note that, subjects within one stage in the four time blocks were the same.

**Table 2:** Results of repeated measures ANOVAs for assessing time-on-task effect on ANS parameters

	Heart rate					Skin conductance level				
	Time block (min)				Effect (η_p_^2^)	Time block (min)				Effect (η_p_^2^)
	1–30	31–60	61–90	91–120		1–30	31–60	61–90	91–120	
*Ignored condition* ^a^										
A1	65.63 (7.38)	64.04 (7.60)	63.97 (7.59)	64.52 (7.78)	F_3,63_ = 5.370** (0.204)	3.75 (4.40)	3.81 (4.82)	4.02 (4.73)	4.37 (5.97)	F_1.183,24.852_ = 0.472 (0.022)
A2	62.30 (6.96)	60.74 (7.73)	60.90 (7.31)	61.91 (8.20)	F_3,42_ = 1.574 (0.101)	3.78 (4.80)	3.57 (5.39)	3.84 (5.12)	4.65 (7.13)	F_1.124,15.742_ = 0.493 (0.034)
B1	61.79 (6.85)	60.26 (6.96)	60.18 (7.76)	60.97 (8.01)	F_3,105_ = 3.381* (0.088)	2.77 (3.26)	2.68 (3.21)	2.94 (3.53)	3.55 (5.22)	F_1.153,40.351_ = 2.263 (0.061)
B2/3	61.42 (6.61)	59.87 (6.52)	59.52 (6.75)	60.89 (8.36)	F_1.981,45.573_ = 2.314 (0.091)	2.31 (3.27)	2.33 (3.56)	2.59 (3.60)	2.78 (3.68)	F_1.725,39.682_ = 2.764 (0.107)
*Attended condition* ^b^										
A1	65.76 (9.49)	65.65 (9.55)	65.37 (9.18)	65.21 (9.10)	F_2.395,74.258_ = 0.493 (0.016)	2.66 (1.72)	2.54 (2.35)	2.76 (3.15)	2.66 (2.29)	F_1.366,42.343_ = 0.208 (0.007)
B1	63.45 (9.50)	62.16 (9.01)	61.65 (9.37)	61.81 (8.78)	F_2.023,66.757_ = 5.509** (0.143)	2.51 (1.48)	2.41 (1.95)	2.70 (3.04)	2.81 (2.68)	F_1.280,42.247_ = 0.746 (0.022)
B2/3	60.00 (8.08)	58.71 (7.31)	58.27 (7.67)	59.21 (8.45)	F_3,54_ = 3.059* (0.145)	2.03 (1.20)	1.86 (1.06)	1.78 (0.92)	1.85 (0.96)	F_1.378,24.810_ = 1.323 (0.068)

Standard deviations are presented in parentheses

*p < .05; **p < .01; ***p < .001

^a^The sample sizes in examination of time-on-task effect in the ignored condition during certain stage were different: n = 22 in stage A1, n = 15 in stage A2, n = 36 in stage B1, n = 24 in stage B2/3

^b^The sample sizes in examination time-on-task effect in the attended condition during certain stage were different: n = 32 in stage A1, n = 34 in stage B1, n = 19 in stage B2/3

*In the ignored condition*, rmANOVAs revealed a significant main effect of *time*-*on*-*task* in stages A1 (*p *= .002) and B1 (*p *= .021) for HR, explaining about 20% and 10% of variance in stage A1 and B1, respectively (see Table 2). No significant *time*-*on*-*task* effect was found for SCL in any of the stages (.082 ≤ *p* ≤ .530).

For stage A1, HR was significantly higher in min 1–30 relative to min 61–90 (mean difference = 1.658, *p *= .022). For stage B1, HR in min 1–30 was significantly higher than in min 31–60 (mean difference = 1.532, *p *= .037).

*In the attended condition*, a significant main effect of *time*-*on*-*task* was obtained in stages B1 (*p *= .006) and B2/3 (*p *= .036) for HR, whereas no significant *time*-*on*-*task* effect was found for SCL (.274 ≤ *p* ≤ .726). 10% of the variance in HR in stages B1 and B2/3 was explained by the determined effect, whereas it explained only 2% of the variance in stage A1 (see Table 2).

For stage B1, HR in min 1–30 was significantly higher relative to min 31–60 (mean difference = 1.296, *p *= .002) and min 61–90 (mean difference = 1.802, *p *= .023). For stage B2/3, HR in min 1–30 was significantly higher when compared to min 61–90 (mean difference = 1.735, *p *= .042).

### HR and SCL between EEG-vigilance stages during each time block

The detailed results from the analyses of HR and SCL across EEG-vigilance stages within each time block in the ignored and attended condition, as well as their summaries are provided in Additional file 4.

#### Comparisons in the ignored condition

The ANS differences in stage A1 versus A2/A3 were weakly pronounced in the first block, but present in most cases in the last three blocks. The differences between stages A2 and A3 in all blocks could not be confirmed, due to either non-significant results or insufficient sample sizes. The ANS values in stages B1 and B2/3 respectively differed significantly from other higher vigilance stages in most available cases in all blocks; and in most cases B1 and B2/3 also differed significantly from each other. Few comparisons with stage C could be conducted due to the rare occurrence of stage C. However, the ANS in stage C showed stable differences against all other stages in block min 31–60 and also differed from A1 in all other blocks. The differences between stages 0 versus A1 were only evidenced by SCL in all blocks except for min 61–90.

#### Comparisons in the attended condition

The differences in HR in stage A1 versus A2/A3 were most pronounced in the last two blocks, while SCL differences were obtained in most cases in all four blocks. ANS differences between stages A2 and A3 could not be confirmed in any block. The ANS values in stages B1 and B2/3 consistently differed from stages 0 and A1 in most cases in all blocks, but did not differ from other higher stages. Significant differences between stages B1 and B2/3 were consistently present in most cases. Comparisons concerning stage C were limited in most cases, except in block min 31–60 and min 61–90, wherein ANS differed significantly from stages A1 and B1. The differences between stages 0 versus A1 were only evidenced by SCL in the first two blocks.

## Discussion

In the present study we examined ANS activity in different states of brain arousal (indexed by EEG-vigilance stages) in ignored and attended conditions of an auditory oddball task of a 2-h EEG. We examined the association of brain arousal and ANS activity within four subsequent 30-min time blocks to control for time-on-task (Hypothesis 1). We additionally examined the time-on-task effect on ANS activity, restricting analyses to individual EEG-vigilance stages (Hypothesis 2).

In line with Hypothesis 1, we found a clear decrease of HR and SCL from EEG-vigilance stage A1 to C over the entire 2-h EEG recording. This finding is in line with the study findings from Olbrich et al. [11] and more recently by Jawinski et al. [28], who demonstrated a gradual change of ANS activity in different states of brain arousal in 15-min resting EEG data. Most importantly, these gradual ANS changes were present in most cases within each 30-min time block. These findings demonstrate that changes in ANS activity correspond to declines in EEG-vigilance stages from A1 to C, even when controlling for time-on-task. The present study demonstrates this association for the first time under oddball conditions and therefore provides further validation of VIGALL 2.1.

**Fig. 3:**
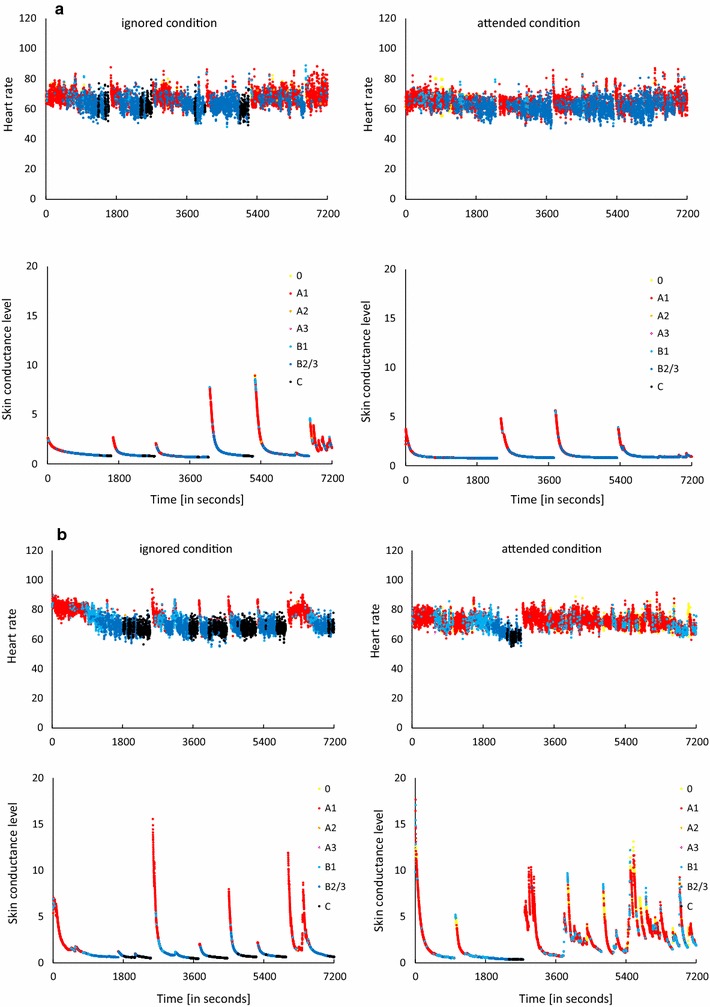
HR and SCL for two individuals (**a**, **b**) over 2-h EEG. Each dot represents the corresponding ANS value in 1 s. The visible gaps between dots indicate possible wake-up reaction. After each wake-up, however, EEG-vigilance stage A1 (red dots) rather than stage 0 (yellow dots) occurred almost exclusively. Subsequently a steep decrease occurred in about 100 s but without change in level of brain arousal

In contrast, an unexpectedly lower HR and SCL in stage 0 (associated with cognitively active wakefulness) as compared to stage A1 (associated with relaxed wakefulness) was obtained over the 2-h recording time in both the ignored and attended conditions. This was also the case for SCL in all 30-min time blocks and in the most cases for HR, except for the second time block in the attended condition. In the Olbrich et al. [11] study, in which similar results were found using an earlier VIGALL version, the authors attributed this finding to the discriminative validity of EEG-vigilance stages 0 versus B1. Although 0/B1 separation has been improved upon in more recent VIGALL versions, this distinction remains a challenge, as both stages are characterized by desynchronized non alpha EEG via VIGALL (see Table 1). We also cannot rule out a possible 0/B1 misclassification in the present study. However, the difference obtained between stages 0 and A1 could be attributed to the wake-up reactions introduced by the experimenter. To acquire sufficient variability in EEG-vigilance, study participants were woken up 5-min after sleep-onset. To obtain a detailed assessment of individual temporal alterations in brain arousal and ANS activity, we plotted HR and SCL values with a resolution of 1 s for each subject in the ignored and the attended conditions, indicating different EEG-vigilance stages with colored points (see Fig. 3a, b). The wake-up reaction was characterized by a short episode of elevated HR and SCL values and during this episode stage A1 occurred almost exclusively. The HR and SCL during this episode were much higher than the HR and SCL in stage 0 and therefore led to an overall higher ANS value in stage A1 than in stage 0.

The simultaneous increase in cortical and ANS arousal during the wake-up reaction may reflect a protective mechanism to potential threatening stimuli [29]. Following this reaction, a subsequent steep decline in ANS activity in the absence of simultaneous cortical decline was observed in our healthy subjects. This is understood as a normal physiological return to baseline level (pattern: rapid decline of ANS but delayed decline of cortical arousal) as the experiment instructions necessitated. It would be interesting to examine whether there are similar patterns in clinical populations, for example in patients with depression or ADHD.

Hypothesis 2 was confirmed for HR. A strength of the current study is the extended recording period of 2 h compared to 15 min in prior work [11]. This allowed us to assess the direct time-on-task effect in the same subjects and to restrict the analyses to particular EEG-vigilance stages. Post hoc tests showed that the time-on-task effect was most evident between the first and all subsequent blocks. Regarding the direction of the change between the first and subsequent blocks, a decrease in HR in both the ignored and attended conditions was seen, although brain arousal was constant. One reason for this finding could be inherent in VIGALL’s Standard Operating Procedure. At the beginning of the recording, the subject’s body position was changed from an upright to a semi-supine position and individuals were asked to close their eyes [7]. This postural change may have induced an increase in cutaneous blood flow due to a deactivation of sympathetic vasoconstriction reflexes, resulting in reduced sympathetic outflow and decrease in HR [30]. In addition, the semi-supine position is associated with a considerable increase in venous return from the extremities to the heart, minimizing the effort against gravity which then consequently results in down-regulated HR [31]. The effect of postural change on brain arousal [32–34] and ANS activity [33, 35] has been evidenced in several studies, which may indicate the possible confounding effect of postural change in examinations requiring change body positions such as in fMRI studies.

Interestingly, concerning the time-on-task effect on HR in stage A1, we observed a clear difference—about 18% in explained variance—between the ignored and attended conditions. This finding illustrates the influence of a cognitive task on HR within a waking state, where most stimuli are perceived and a behavioral response must be maintained [24]. However, the influence of the cognitive task on HR became smaller upon occurrence of the drowsy state.

Our results underscore that the waking state is, at the physiological level, not a uniform state. Study participants displayed broad variability in EEG-vigilance stages during both conditions of the oddball task. Thus, in study designs where attention and cognitive processing are crucial, controlling for EEG-vigilance stages seems important, because arousal states influence performance, for example, reaction times [24]. This could also have implications for creating apps or devices for real-world situations such as commercial driving.

As a limitation of this study, we did not control for changes in temperature in the booth during the recording period, which might have resulted in increased temperature and humidity and therefore contributed to SCL [36]. The lack of time-on-task effect on SCL may be due to this environmental factor. Our finding of a numeric increase in SCL values across time blocks (Fig. 2) may therefore be attributed to the increased temperature in the booth (4–5 m^2^) over 2 h recording.

A second limitation is the individual difference in arousal variability across the EEG recording and a minimum criterion of 10 epochs for every subject in EEG-vigilance stages. Because sufficient sample sizes were not obtained for each time block, some comparisons could not be made (e.g. stage A3 vs. C). This was especially pronounced in the attended condition. Because subjects were performing a task, they fell asleep less frequently and more often stayed awake, which resulted in more frequent higher EEG-vigilance stages in the attended condition. Further, we acknowledge that the relatively small and selected sample limits the generalizability of our findings.

## Conclusion

In conclusion, this is the first study to directly determine a time-on-task effect on HR when restricting analyses to particular states of brain arousal. Concurrent changes in ANS activity and EEG-vigilance were found over the 2-h recording period and in most cases within each 30-min time block, contributing to the validation of VIGALL 2.1.

## Additional files

**Additional file 1.** Description of EEG preprocessing.

**Additional file 2.** Percentages of EEG-vigilance stages in the ignored and attended condition.

**Additional file 3.** Results of pared sample *t* tests for ANS parameters between different EEG-vigilance stages in the ignored and attended condition.

**Additional file 4.** Summaries and detailed results of pared sample *t* tests for heart rate and skin conductance level between different EEG-vigilance stages in corresponding time block in ignored and attended condition.

## Abbreviations

EEG: electroencephalography
VIGALL: Vigilance Algorithm Leipzig
EOG: electrooculogram
ANS: autonomic nervous system
HR: heart rate
SCL: skin conductance level
RDoC: Research Domain Criteria Project
ADHD: attention-deficit/hyperactivity disorder
ECG: electrocardiogram
rmANOVA: repeated measures analyses of variance
