# Supplementary material for: Impact of brain arousal and time-on-task on autonomic nervous system activity in the wake-sleep transition
Source: BMC Neurosci. 2018 Apr 11;19:18. doi: 10.1186/s12868-018-0419-y (PMC5896037; doi:10.1186/s12868-018-0419-y)
Supplement: Supplementary file 1 — Additional file 1. Description of EEG preprocessing. [file 12868_2018_419_MOESM1_ESM.docx]

**EEG preprocessing**

EEG preprocessing was done applying Vision Analyzer (Brain Products GmbH, Gilching, Germany) with the following steps:

1) An offline bandpass filter between 0.5-70Hz (with notch-filter at 50Hz) was applied to the EEG and EOG data, with exception that there was no high-pass filter for EOG.

2) The 2-h EEG data was then divided into equal 1-s segments.

3) Very obvious muscle, swallowing, eye movement and sweating artifacts were marked when screening for rough visual artifacts.

4) Thereafter, an independent components analysis was performed. The eye movement and continuous muscle artifacts were removed by extracting independent components that clearly contained only artifact-related information.

5) The EEG segments were again visually screened in-depth for the remaining artifacts that were not removed by rough artifact screening and ICA.

6) The classification of EEG-vigilance stage C via VIGALL relies on the occurrence of graph elements indicating sleep onset (i.e. K-complex or sleep spindles), therefore all EEGs had visually been screened for such graph elements and the respective segments had been manually marked. This step can also be performed before step 4.

7) In order to increase processing speed, the sampling rate was changed to 100 Hz.

8) Afterwards, the already segmented epochs in step 2 were reversed because VIGALL requires a continuous EEG.
