# Supplementary material for: Impact of brain arousal and time-on-task on autonomic nervous system activity in the wake-sleep transition
Source: BMC Neurosci. 2018 Apr 11;19:18. doi: 10.1186/s12868-018-0419-y (PMC5896037; doi:10.1186/s12868-018-0419-y)
Supplement: Supplementary file 2 — Additional file 2. Percentages of EEG-vigilance stages in the ignored and attended condition. [file 12868_2018_419_MOESM2_ESM.docx]

**Table S1. Percentages of EEG-vigilance stages in ignored and attended condition**

|  |  | ignored condition | | |  | attended condition | | |
| --- | --- | --- | --- | --- | --- | --- | --- | --- |
| Block | EEG-vigilance stage | n | mean (%) | SD |  | n | mean (%) | SD |
| overall  2h | 0 | 35 | 4.38 | 5.73 |  | 34 | 6.75 | 10.51 |
|  | A1 | 39 | 54.17 | 19.31 |  | 39 | 50.54 | 19.11 |
|  | A2 | 30 | 2.33 | 4.01 |  | 24 | 2.54 | 7.23 |
|  | A3 | 23 | 1.75 | 4.26 |  | 24 | 1.62 | 5.38 |
|  | B1 | 39 | 21.65 | 14.42 |  | 38 | 22.61 | 15.76 |
|  | B2/3 | 36 | 11.05 | 10.80 |  | 37 | 13.10 | 16.86 |
|  | C | 30 | 4.63 | 6.42 |  | 21 | 2.80 | 4.47 |
| time block  min 1-30 | 0 | 28 | 4.94 | 7.16 |  | 33 | 8.01 | 12.45 |
|  | A1 | 32 | 39.56 | 26.21 |  | 35 | 45.26 | 25.76 |
|  | A2 | 18 | 2.60 | 4.98 |  | 14 | 2.44 | 6.53 |
|  | A3 | 18 | 1.85 | 4.40 |  | 12 | 1.29 | 4.53 |
|  | B1 | 39 | 22.49 | 17.92 |  | 37 | 23.79 | 18.80 |
|  | B2/3 | 30 | 9.26 | 11.05 |  | 26 | 9.14 | 13.87 |
|  | C | 14 | 3.52 | 6.71 |  | 7 | 1.03 | 2.66 |
| time block min 31-60 | 0 | 24 | 3.30 | 5.42 |  | 24 | 4.01 | 6.80 |
|  | A1 | 37 | 41.44 | 21.47 |  | 38 | 40.25 | 20.83 |
|  | A2 | 20 | 2.08 | 3.66 |  | 12 | 2.50 | 8.03 |
|  | A3 | 17 | 1.70 | 3.87 |  | 11 | 1.71 | 6.36 |
|  | B1 | 39 | 25.55 | 17.03 |  | 37 | 26,77 | 19.21 |
|  | B2/3 | 33 | 15.01 | 14.15 |  | 33 | 17.33 | 20.92 |
|  | C | 25 | 6.16 | 8.67 |  | 13 | 4.80 | 8.79 |
| time block min 61-90 | 0 | 25 | 4.26 | 5.42 |  | 26 | 6.40 | 13.03 |
|  | A1 | 31 | 35.81 | 25.05 |  | 37 | 44.95 | 22.00 |
|  | A2 | 19 | 2.46 | 4.70 |  | 12 | 2.44 | 7.76 |
|  | A3 | 13 | 1.79 | 4.93 |  | 14 | 1.69 | 5.29 |
|  | B1 | 37 | 20.07 | 14.47 |  | 35 | 21.46 | 17.85 |
|  | B2/3 | 30 | 11.19 | 13.62 |  | 29 | 14.86 | 19.43 |
|  | C | 13 | 6.19 | 11.29 |  | 15 | 3.15 | 5.93 |
| time block  min 91-120 | 0 | 28 | 4.74 | 7.30 |  | 27 | 8.19 | 12.90 |
|  | A1 | 30 | 39.66 | 27.63 |  | 36 | 47.75 | 23.10 |
|  | A2 | 18 | 1.99 | 3.51 |  | 13 | 2.45 | 7.42 |
|  | A3 | 13 | 1.51 | 4.19 |  | 11 | 1.48 | 5.58 |
|  | B1 | 36 | 18.80 | 14.98 |  | 37 | 19.04 | 16.14 |
|  | B2/3 | 29 | 9.37 | 12.26 |  | 25 | 11.45 | 17.76 |
|  | C | 11 | 2.79 | 5.71 |  | 9 | 2.48 | 6.04 |

n shows the number of subjects reaching the criterion of at least 10 epochs in each EEG-vigilance stage.

SD=standard deviation
