# Supplementary material for: Impact of brain arousal and time-on-task on autonomic nervous system activity in the wake-sleep transition
Source: BMC Neurosci. 2018 Apr 11;19:18. doi: 10.1186/s12868-018-0419-y (PMC5896037; doi:10.1186/s12868-018-0419-y)
Supplement: Supplementary file 3 — Additional file 3. Results of pared sample t tests for ANS parameters between different EEG-vigilance stages in the ignored and attended condition. [file 12868_2018_419_MOESM3_ESM.docx]

**Table S2. Results of paired sample t-tests for ANS parameters between different EEG-vigilance stages in the ignored condition**

|  | | heart rate | | | |  | skin conductance level | | | |
| --- | --- | --- | --- | --- | --- | --- | --- | --- | --- | --- |
|  |  |  | effect | | |  |  | effect | | |
| comparison | | mean (SD) | df | t | *p* |  | mean (SD) | df | t | *p* |
| 0 | vs. A1 | 63.60 (8.32)  64.53 (7.40) | 34 | -1.926 | .063 |  | -0.04 (0.42)  0.29 (0.30) | 34 | -4.800 | 3.12E-5 |
|  | vs. A2 | 62.30 (7.65)  62.51 (6.78) | 26 | -0.377 | .709 |  | -0.06 (0.44)  -0.13 (0.62) | 26 | -1.916 | .066 |
|  | vs. A3 | 62.73 (9.07)  62.71 (7.49) | 20 | 1.159 | .260 |  | 0.00 (0.49)  -0.18 (0.40) | 20 | 1.313 | .204 |
|  | vs. B1 | 63.60 (8.32)  59.92 (6.81) | 34 | 6.023 | 8.05E-7 |  | -0.04 (0.42)  -0.28 (0.34) | 34 | 2.972 | .005 |
|  | vs. B2/3 | 63.50 (8.43)  58.29 (5.95) | 31 | 6.933 | 8.91E-8 |  | -0.03 (0.44)  -0.49 (0.36) | 31 | 4.122 | 2.60E-4 |
|  | vs. C | 62.95 (8.88)  56.97 (6.22) | 26 | 6.783 | 3.37E-7 |  | -0.05 (0.45)  -0.73 (0.35) | 26 | 6.162 | 1.62E-6 |
| A1 | vs. A2 | 63.53 (7.22)  62.67 (7.00) | 29 | 3.898 | .001 |  | 0.27 (0.28)  0.12 (0.60) | 29 | 1.543 | .134 |
|  | vs. A3 | 64.62 (8.49)  62.73 (7.72) | 22 | 3.972 | .001 |  | 0.31 (0.32)  -0.18 (0.38) | 22 | 4.230 | 3.44E-4 |
|  | vs. B1 | 65.11 (8.14)  60.31 (7.25) | 38 | 11.000 | 2.26E-13 |  | 0.29 (0.29)  -0.29 (0.33) | 38 | 8.081 | 8.93E-10 |
|  | vs. B2/3 | 65.09 (8.18)  58.80 (6.53) | 35 | 11.609 | 1.49E-13 |  | 0.30 (0.29)  -0.50 (0.34) | 35 | 8.936 | 1.48E-10 |
|  | vs. C | 65.13 (8.59)  57.71 (6.40) | 29 | 10.856 | 9.91E-12 |  | 0.35 (0.30)  -0.74 (0.34) | 29 | 12.787 | 1.91E-13 |
| A2 | vs. A3 | 63.03 (8.21)  62.08 (7.77) | 20 | 2.472 | .023 |  | 0.20 (0.68)  -0.16 (0.39) | 20 | 2.543 | .019 |
|  | vs. B1 | 62.67 (7.00)  58.80 (6.40) | 29 | 8.449 | 2.61E-9 |  | 0.12 (0.60)  -0.31 (0.34) | 29 | 4.455 | 1.15E-4 |
|  | vs. B2/3 | 62.92 (7.18)  57.83 (6.10) | 27 | 8.768 | 2.20E-9 |  | 0.14 (0.61)  -0.48 (0.36) | 27 | 4.681 | 7.17E-5 |
|  | vs. C | 62.88 (7.50)  56.91 (6.53) | 22 | 11.988 | 4.05E-11 |  | 0.21 (0.65)  -0.74 (0.34) | 22 | 6.672 | 1.05E-6 |
| A3 | vs. B1 | 62.73 (7.72)  59.33 (7.64) | 22 | 7.223 | 3.08E-7 |  | -0.18 (0.38)  -0.29 (0.38) | 22 | 1.469 | .156 |
|  | vs. B2/3 | 62.73 (7.72)  58.33 (6.88) | 22 | 6.628 | 1.15E-6 |  | -0.18 (0.38)  -0.49 (0.36) | 22 | 4.542 | 1.61E-4 |
|  | vs. C | 62.46 (7.95)  57.05 (7.05) | 18 | 9.315 | 2.63E-8 |  | -0.16 (0.37)  -0.75 (0.32) | 18 | 7.022 | 1.49E-6 |
| B1 | vs. B2/3 | 60.00 (7.18)  58.80 (6.53) | 35 | 2.793 | .008 |  | -0.31 (0.32)  -0.50 (0.34) | 35 | 3.205 | .003 |
|  | vs. C | 59.65 (7.19)  57.71 (6.40) | 29 | 3.908 | .001 |  | -0.31 (0.34)  -0.74 (0.34) | 29 | 6.907 | 1.37E-7 |
| B2/3 | vs. C | 58.45 (6.45)  57.71 (6.40) | 29 | 1.681 | .103 |  | -0.52 (0.33)  -0.74 (0.34) | 29 | 5.026 | 2.36E-5 |

SD=standard deviation

df=degrees of freedom

**Table S3. Results of paired sample t-tests for ANS parameters between different EEG-vigilance stages in the attended condition**

|  | | heart rate | | | |  | skin conductance level | | | |
| --- | --- | --- | --- | --- | --- | --- | --- | --- | --- | --- |
|  |  |  | effect | | |  |  | effect | | |
| comparison | | mean (SD) | df | t | *p* |  | mean (SD) | df | t | *p* |
| 0 | vs. A1 | 64.67 (8.95)  65.12 (8.87) | 33 | -1.051 | .301 |  | 0.16 (0.46)  0.25 (0.32) | 33 | -1.080 | .288 |
|  | vs. A2 | 65.23 (10.25)  64.81 (10.04) | 18 | 0.588 | .564 |  | 0.10 (0.43)  -0.05 (0.30) | 18 | 1.702 | .106 |
|  | vs. A3 | 65.94 (10.36)  64.61 (9.97) | 19 | 1.458 | .161 |  | 0.18 (0.46)  -0.21 (0.35) | 19 | 3.695 | .002 |
|  | vs. B1 | 64.67 (8.95)  61.96 (8.81) | 33 | 6.084 | 7.54E-7 |  | 0.16 (0.46)  -0.20 (0.26) | 33 | 4.165 | 2.10E-4 |
|  | vs. B2/3 | 64.81 (9.00)  60.05 (8.07) | 31 | 7.258 | 3.64E-8 |  | 0.17 (0.47)  -0.41 (0.40) | 31 | 4.717 | 4.82E-5 |
|  | vs. C | 64.54 (8.87)  57.33 (7.61) | 17 | 7.295 | 1.25E-6 |  | 0.26 (0.49)  -0.78 (0.59) | 17 | 5.052 | 9.82E-5 |
| A1 | vs. A2 | 64.92 (10.39)  63.94 (10.19) | 23 | 4.262 | 2.93E-4 |  | 0.14 (0.15)  -0.04 (0.29) | 23 | 2.568 | .017 |
|  | vs. A3 | 65.01 (10.52)  63.24 (10.00) | 23 | 4.002 | .001 |  | 0.21 (0.34)  -0.21 (0.33) | 23 | 3.774 | .001 |
|  | vs. B1 | 64.45 (9.18)  61.23 (9.04) | 37 | 9.003 | 6.80E-11 |  | 0.24 (0.30)  -0.21 (0.27) | 37 | 6.252 | 2.86E-7 |
|  | vs. B2/3 | 64.67 (9.15)  59.80 (8.22) | 36 | 8.964 | 1.06E-10 |  | 0.24 (0.31)  -0.39 (0.40) | 36 | 6.253 | 3.19E-7 |
|  | vs. C | 64.49 (9.14)  56.85 (7.26) | 20 | 8.959 | 1.94E-8 |  | 0.30 (0.39)  -0.82 (0.60) | 20 | 6.534 | 2.28E-6 |
| A2 | vs. A3 | 63.69 (10.54)  62.81 (10.12) | 20 | 2.886 | 0.09 |  | -0.07 (0.29)  -0.18 (0.34) | 20 | 2.256 | .035 |
|  | vs. B1 | 63.67 (10.33)  61.06 (10.14) | 22 | 5.870 | 6.60E-6 |  | -0.05 (0.29)  -0.17 (0.29) | 22 | 1.920 | .068 |
|  | vs. B2/3 | 63.96 (10.32)  60.25 (9.32) | 21 | 4.997 | 6.04E-5 |  | -0.04 (0.28)  -0.31 (0.38) | 21 | 2.793 | .011 |
|  | vs. C | 63.93 (10.54)  57.79 (8.62) | 11 | 4.829 | .001 |  | -0.04 (0.35)  -0.89 (0.52) | 11 | 5.277 | 2.62E-4 |
| A3 | vs. B1 | 63.02 (10.17)  61.07 (10.50) | 22 | 3.733 | .001 |  | -0.22 (0.33)  -0.18 (0.30) | 22 | -0.521 | .607 |
|  | vs. B2/3 | 63.40 (10.20)  60.11 (9.69) | 21 | 4.577 | 1.64E-4 |  | -0.19 (0.33)  -0.35 (0.41) | 21 | 1.439 | .165 |
|  | vs. C | 62.86 (9.60)  57.62 (8.46) | 13 | 4.675 | 4.35E-4 |  | -0.18 (0.37)  -0.85 (0.44) | 13 | 4.493 | .001 |
| B1 | vs. B2/3 | 61.25 (9.13)  59.45 (8.06) | 35 | 3.879 | 4.43E-4 |  | -0.19 (0.27)  -0.40 (0.39) | 35 | 3.815 | .001 |
|  | vs. C | 60.77 (9.02)  56.85 (7.26) | 20 | 5.243 | 3.95E-5 |  | -0.18 (0.29)  -0.82 (0.60) | 20 | 5.802 | 1.12E-5 |
| B2/3 | vs. C | 58.05 (7.31)  56.85 (7.26) | 20 | 2.512 | .021 |  | -0.54 (0.41)  -0.82 (0.60) | 20 | 3.730 | .001 |

SD=standard deviation

df=degrees of freedom
