# Supplementary material for: Impact of brain arousal and time-on-task on autonomic nervous system activity in the wake-sleep transition
Source: BMC Neurosci. 2018 Apr 11;19:18. doi: 10.1186/s12868-018-0419-y (PMC5896037; doi:10.1186/s12868-018-0419-y)
Supplement: Supplementary file 4 — Additional file 4. Summaries and detailed results of pared sample t tests for heart rate and skin conductance level between different EEG-vigilance stages in corresponding time block in ignored and attended condition. [file 12868_2018_419_MOESM4_ESM.docx]

**Table S4. Summary of results for ANS parameters between EEG-vigilance stages within each block in the ignored condition**

| time block | EEG-vigilance stage | heart rate | | | | | |  | skin conductance level | | | | | |
| --- | --- | --- | --- | --- | --- | --- | --- | --- | --- | --- | --- | --- | --- | --- |
|  |  | 0 | A1 | A2 | A3 | B1 | B2/3 |  | 0 | A1 | A2 | A3 | B1 | B2/3 |
| min 1-30 | A1 | n.s. |  |  |  |  |  |  | ** |  |  |  |  |  |
|  | A2 | n.s. | n.s. |  |  |  |  |  | n.s. | n.s. |  |  |  |  |
|  | A3 | n.s. | n.s. | n.s. |  |  |  |  | n.s. | ** | ** |  |  |  |
|  | B1 | *** | *** | *** | ** |  |  |  | *** | *** | *** | n.s. |  |  |
|  | B2/3 | ** | *** | ** | ** | n.s. |  |  | ** | *** | ** | * | * |  |
|  | C | - | *** | - | - | n.s. | n.s. |  | - | *** | - | - | *** | ** |
| min 31-60 | A1 | n.s. |  |  |  |  |  |  | * |  |  |  |  |  |
|  | A2 | - | ** |  |  |  |  |  | - | ** |  |  |  |  |
|  | A3 | - | ** | n.s. |  |  |  |  | - | ** | n.s. |  |  |  |
|  | B1 | *** | *** | *** | *** |  |  |  | n.s. | *** | * | * |  |  |
|  | B2/3 | *** | *** | *** | *** | * |  |  | ** | *** | ** | *** | ** |  |
|  | C | *** | *** | *** | *** | ** | ** |  | ** | *** | *** | *** | *** | ** |
| min 61-90 | A1 | n.s. |  |  |  |  |  |  | n.s. |  |  |  |  |  |
|  | A2 | - | * |  |  |  |  |  | - | ** |  |  |  |  |
|  | A3 | - | * | n.s. |  |  |  |  | - | *** | n.s. |  |  |  |
|  | B1 | ** | *** | *** | *** |  |  |  | ** | *** | ** | * |  |  |
|  | B2/3 | ** | *** | ** | ** | * |  |  | ** | *** | ** | * | ** |  |
|  | C | - | *** | - | - | ** | n.s. |  | - | *** | - | - | ** | n.s. |
| min 91-120 | A1 | n.s. |  |  |  |  |  |  | ** |  |  |  |  |  |
|  | A2 | n.s. | * |  |  |  |  |  | n.s. | n.s. |  |  |  |  |
|  | A3 | - | * | - |  |  |  |  | - | * | - |  |  |  |
|  | B1 | *** | *** | ** | ** |  |  |  | ** | *** | n.s. | n.s. |  |  |
|  | B2/3 | *** | *** | * | * | * |  |  | ** | *** | n.s. | * | ** |  |
|  | C | - | *** | - | - | n.s. | n.s. |  | - | *** | - | - | ** | ** |

* *p*<.05 ** *p*<.01 *** *p*<.001

n.s.=not significant

- The tests were not executed due to insufficient sample size (n≤10).

**Table S5. Summary of results for ANS parameters between EEG-vigilance stages within each block in the attended condition**

| time block | EEG-vigilance stage | heart rate | | | | | |  | skin conductance level | | | | | |
| --- | --- | --- | --- | --- | --- | --- | --- | --- | --- | --- | --- | --- | --- | --- |
|  |  | 0 | A1 | A2 | A3 | B1 | B2/3 |  | 0 | A1 | A2 | A3 | B1 | B2/3 |
| min 1-30 | A1 | n.s. |  |  |  |  |  |  | * |  |  |  |  |  |
|  | A2 | n.s. | n.s. |  |  |  |  |  | n.s. | * |  |  |  |  |
|  | A3 | - | n.s. | n.s. |  |  |  |  | - | ** | * |  |  |  |
|  | B1 | *** | *** | ** | - |  |  |  | *** | *** | * | - |  |  |
|  | B2/3 | *** | *** | - | - | * |  |  | *** | *** | - | - | * |  |
|  | C | - | - | - | - | - | - |  | - | - | - | - | - | - |
| min 31-60 | A1 | n.s. |  |  |  |  |  |  | ** |  |  |  |  |  |
|  | A2 | - | n.s. |  |  |  |  |  | - | n.s. |  |  |  |  |
|  | A3 | - | * | - |  |  |  |  | - | * | - |  |  |  |
|  | B1 | *** | *** | - | - |  |  |  | n.s. | *** | - | - |  |  |
|  | B2/3 | *** | *** | - | - | * |  |  | n.s. | *** | - | - | * |  |
|  | C | - | *** | - | - | * | n.s. |  | - | *** | - | - | * | * |
| min 61-90 | A1 | n.s. |  |  |  |  |  |  | n.s. |  |  |  |  |  |
|  | A2 | - | ** |  |  |  |  |  | - | n.s. |  |  |  |  |
|  | A3 | - | *** | n.s. |  |  |  |  | - | * | n.s. |  |  |  |
|  | B1 | *** | *** | - | ** |  |  |  | * | *** | - | n.s. |  |  |
|  | B2/3 | ** | *** | - | * | * |  |  | * | *** | - | n.s. | * |  |
|  | C | - | *** | - | - | * | n.s. |  | - | *** | - | - | * | n.s. |
| min 91-120 | A1 | n.s. |  |  |  |  |  |  | n.s. |  |  |  |  |  |
|  | A2 | - | ** |  |  |  |  |  | - | * |  |  |  |  |
|  | A3 | - | ** | n.s. |  |  |  |  | - | * | n.s. |  |  |  |
|  | B1 | * | *** | * | - |  |  |  | * | *** | n.s. | - |  |  |
|  | B2/3 | * | *** | - | - | * |  |  | * | *** | - | - | n.s. |  |
|  | C | - | - | - | - | - | - |  | - | - | - | - | - | - |

* *p*<.05 ** *p*<.01 *** *p*<.001

n.s.=not significant

- The tests were not executed due to insufficient sample size (n≤10).

**Table S6. Results of paired sample t-tests for heart rate and skin conductance level between different EEG-vigilance stages in time block min 1-30 in the ignored condition**

| ignored condition | | heart rate | | | |  | skin conductance level | | | |
| --- | --- | --- | --- | --- | --- | --- | --- | --- | --- | --- |
|  |  |  | effect | | |  |  | effect | | |
| comparison | | mean (SD) | df | t | *p* |  | mean (SD) | df | t | *p* |
| 0 | vs. A1 | 64.04 (8.33)  64.82 (7.85) | 21 | -1.948 | .065 |  | 0.04 (0.53)  0.45 (0.44) | 21 | -3.976** | .001 |
|  | vs. A2 | 59.49 (7.79)  60.54 (6.86) | 12 | -1.796 | .098 |  | -0.14 (0.24)  0.03 (0.38) | 12 | -1.343 | .204 |
|  | vs. A3 | 60.92 (8.22)  61.16 (7.38) | 13 | -0.306 | .765 |  | 0.08 (0.65)  -0.30 (0.38) | 13 | 1.737 | .106 |
|  | vs. B1 | 63.06 (8.13)  60.89 (7.16) | 27 | 4.248*** | 2.29E-4 |  | -0.06 (0.52  -0.42 (0.31) | 27 | 5.009*** | 2.97E-5 |
|  | vs. B2/3 | 62.48 (8.75)  59.43 (6.66) | 19 | 4.141** | .001 |  | -0.05 (0.57)  -0.57 (0.38) | 19 | 3.486** | .002 |
|  | vs. C^a^ | 62.15 (7.05)  58.26 (6.28) |  |  |  |  | 0.15 (0.82)  -0.82 (0.22) |  |  |  |
| A1 | vs. A2 | 63.45 (8.96)  63.08 (9.05) | 13 | 0.854 | .409 |  | 0.39 (0.39)  0.19 (0.38) | 13 | 1.540 | .148 |
|  | vs. A3 | 64.10 (8.49)  63.17 (9.26) | 15 | 1.460 | .165 |  | 0.48 (0.54)  -0.24 (0.34) | 15 | 4.295** | .001 |
|  | vs. B1 | 66.15 (7.82)  62.42 (7.40) | 31 | 8.948*** | 4.25E-10 |  | 0.47 (0.45)  -0.38 (0.28) | 31 | 10.331*** | 1.46E-11 |
|  | vs. B2/3 | 65.83 (8.29)  60.52 (6.70) | 25 | 8.015*** | 2.27E-8 |  | 0.55 (0.46)  -0.60 (0.32) | 25 | 9.672*** | 6.25E-10 |
|  | vs. C | 65.84 (6.94)  59.62 (5.79) | 13 | 6.325*** | 2.64E-5 |  | 0.77 (0.46)  -0.78 (0.21) | 13 | 12.059*** | 1.97E-8 |
| A2 | vs. A3 | 61.36 (7.79)  60.90 (8.11) | 13 | 1.236 | .238 |  | 0.04 (0.36)  -0.24 (0.36) | 13 | 3.075** | .009 |
|  | vs. B1 | 62.32 (8.08)  59.18 (7.76) | 17 | 5.405*** | 4.74E-5 |  | 0.08 (0.41)  -0.46 (0.28) | 17 | 4.559*** | 2.78E-4 |
|  | vs. B2/3 | 62.30 (8.69)  59.02 (7.79) | 14 | 4.476** | .001 |  | 0.13 (0.42)  -0.54 (0.34) | 14 | 4.150** | .001 |
|  | vs. C^a^ | 62.94 (9.13)  57.94 (7.67) |  |  |  |  | 0.50 (0.37)  -0.86 (0.18) |  |  |  |
| A3 | vs. B1 | 62.81 (8.77)  59.74 (8.12) | 17 | 4.021** | .001 |  | -0.28 (0.34)  -0.41 (0.36) | 17 | 1.255 | .226 |
|  | vs. B2/3 | 62.04 (9.02)  58.71 (7.27) | 15 | 3.584** | .003 |  | -0.28 (0.34)  -0.58 (0.36) | 15 | 2.540* | .023 |
|  | vs. C^a^ | 58.92 (5.45)  55.50 (4.32) |  |  |  |  | -0.21 (0.35)  -0.87 (0.17) |  |  |  |
| B1 | vs. B2/3 | 61.07 (7.16)  60.47 (6.48) | 29 | 1.321 | .197 |  | -0.43 (0.29)  -0.56 (0.36) | 29 | 2.135* | .041 |
|  | vs. C | 61.39 (6.33)  59.62 (5.79) | 13 | 2.152 | .051 |  | -0.40 (0.30)  -0.78 (0.21) | 13 | 5.424*** | 1.16E-4 |
| B2/3 | vs. C | 59.61 (5.50)  59.62 (5.79) | 13 | -0.023 | .982 |  | -0.65 (0 15)  -0.78 (0 21) | 13 | 3.494** | .004 |

^a^ Based on a minimum criterion of 10 epochs, there were no enough subjects (n≤10) in some comparisons

* *p*<.05 ** *p*<.01 *** *p*<.001

SD=standard deviation

df=degrees of freedom

**Table S7. Results of paired sample t-tests for heart rate and skin conductance level between different EEG-vigilance stages in time block min 1-30 in the attended condition**

| attended condition | | heart rate | | | |  | skin conductance level | | | |
| --- | --- | --- | --- | --- | --- | --- | --- | --- | --- | --- |
|  |  |  | effect | | |  |  | effect | | |
| comparison | | mean (SD) | df | t | *p* |  | mean (SD) | df | t | *p* |
| 0 | vs. A1 | 65.70 (9.59)  65.73 (9.42) | 30 | -0.069 | .945 |  | 0.07 (0.55)  0.32 (0.39) | 30 | -2.465* | .020 |
|  | vs. A2 | 63.88 (10.76)  63.23 (10.83) | 11 | 0.880 | .398 |  | 0.04 (0.43)  -0.16 (0.36) | 11 | 1.242 | .240 |
|  | vs. A3 | 65.08 (11.05)  64.15 (11.14) |  |  |  |  | 0.12 (0.43)  -0.28 (0.27) |  |  |  |
|  | vs. B1 | 65.26 (9.46)  63.24 (9.60) | 32 | 4.773*** | 3.84E-5 |  | 0.05 (0.54)  -0.36 (0.27) | 32 | 4.023*** | 3.28E-4 |
|  | vs. B2/3 | 63.99 (9.09)  60.42 (8.57) | 21 | 4.500*** | 1.97E-4 |  | 0.17 (0.59)  -0.63 (0.40) | 21 | 4.709*** | 1.20E-4 |
|  | vs. C^a^ | 61.23 (6.51)  56.73 (5.13) |  |  |  |  | 0.05 (0.76)  -0.93 (0.42) |  |  |  |
| A1 | vs. A2 | 63.82 (10.32)  63.70 (10.74) | 12 | 0.312 | .760 |  | 0.20 (0.17)  -0.10 (0.28) | 12 | 2.546* | .026 |
|  | vs. A3 | 64.99 (10.55)  64.71 (10.94) | 10 | 0.645 | .533 |  | 0.18 (0.18)  -0.21 (0.17) | 10 | 4.800** | .001 |
|  | vs. B1 | 65.12 (9.48)  62.98 (9.77) | 32 | 5.824*** | 1.81E-6 |  | 0.34 (0.39)  -0.35 (0.26) | 32 | 8.286*** | 1.82E-9 |
|  | vs. B2/3 | 63.69 (8.82)  60.34 (8.66) | 24 | 5.511*** | 1.15E-5 |  | 0.39 (0.41)  -0.60 (0.40) | 24 | 7.748*** | 5.54E-8 |
|  | vs. C^a^ | 62.15 (6.69)  56.73 (5.13) |  |  |  |  | 0.69 (0.60)  -0.93 (0.42) |  |  |  |
| A2 | vs. A3 | 64.15 (10.78)  63.81 (10.89) | 11 | 1.532 | .154 |  | -0.12 (0.35)  -0.27 (0.25) | 11 | 2.294* | .042 |
|  | vs. B1 | 63.23 (10.83)  60.78 (10.64) | 11 | 3.879** | .003 |  | -0.16 (0.36)  -0.47 (0.23) | 11 | 2.879* | .015 |
|  | vs. B2/3 | 65.08 (10.59)  61.81 (10.38) |  |  |  |  | -0.09 (0.25)  -0.59 (0.50) |  |  |  |
|  | vs. C^b^ |  |  |  |  |  |  |  |  |  |
| A3 | vs. B1 | 64.15 (11.14)  61.76 (11.16) |  |  |  |  | -0.28 (0.27)  -0.49 (0.24) |  |  |  |
|  | vs. B2/3 | 64.80 (10.75)  61.81 (10.38) |  |  |  |  | -0.22 (0.18)  -0.59 (0.50) |  |  |  |
|  | vs. C^b^ |  |  |  |  |  |  |  |  |  |
| B1 | vs. B2/3 | 60.68 (8.93)  59.70 (8.30) | 24 | 2.152* | .042 |  | -0.41 (0.28)  -0.62 (0.38) | 24 | 2.657* | .014 |
|  | vs. C^a^ | 59.13 (5.33)  56.73 (5.13) |  |  |  |  | -0.25 (0.33)  -0.93 (0.42) |  |  |  |
| B2/3 | vs. C^a^ | 56.77 (4.76)  56.73 (5.13) |  |  |  |  | -0.70 (0.36)  -0.93 (0.42) |  |  |  |

^a^ Based on a minimum criterion of 10 epochs, there were no enough subjects (n≤10) in some comparisons

^b^ These comparisons contained no subjects

* *p*<.05 ** *p*<.01 *** *p*<.001

SD=standard deviation

df=degrees of freedom

**Table S8. Results of paired sample t-tests for heart rate and skin conductance level between different EEG-vigilance stages in time block min 31-60 in the ignored condition**

| ignored condition | | heart rate | | | |  | skin conductance level | | | |
| --- | --- | --- | --- | --- | --- | --- | --- | --- | --- | --- |
|  |  |  | effect | | |  |  | effect | | |
| comparison | | mean (SD) | df | t | *p* |  | mean (SD) | df | t | *p* |
| 0 | vs. A1 | 62.47 (8.51)  62.59 (7.34) | 21 | -0.223 | .826 |  | 0.08 (0.51)  0.39 (0.38) | 21 | -2.519* | .020 |
|  | vs. A2 | 57.60 (4.93)  58.00 (5.00) |  |  |  |  | 0.10 (0.44)  0.10 (0.44) |  |  |  |
|  | vs. A3^a^ | 59.57 (8.24)  58.40 (5.95) |  |  |  |  | 0.23 (0.41)  0.08 (0.33) |  |  |  |
|  | vs. B1 | 62.22 (8.19)  58.84 (6.88) | 23 | 5.155*** | 3.18E-5 |  | 0.08 (0.50)  -0.14 (0.45) | 23 | 1.672 | .108 |
|  | vs. B2/3 | 62.56 (8.84)  57.60 (6.22) | 17 | 5.606*** | 3.15E-5 |  | 0.15 (0.51)  -0.45 (0.36) | 17 | 3.419** | .003 |
|  | vs. C | 62.46 (9.03)  56.42 (5.82) | 13 | 5.808*** | 6.10E-5 |  | 0.17 (0.53)  -0.67 (0.43) | 13 | 4.010** | .001 |
| A1 | vs. A2 | 62.12 (8.10)  61.22 (7.98) | 18 | 3.153** | .006 |  | 0.47 (0.40)  0.05 (0.36) | 18 | 3.800** | .001 |
|  | vs. A3 | 62.81 (8.77)  61.60 (7.97) | 15 | 2.990** | .009 |  | 0.50 (0.44)  0.01 (0.32) | 15 | 3.718** | .002 |
|  | vs. B1 | 64.79 (8.29)  60.29 (7.34) | 36 | 10.830*** | 7.08E-13 |  | 0.46 (0.40)  -0.24 (0.43) | 36 | 6.977*** | 3.52E-8 |
|  | vs. B2/3 | 64.99 (8.43)  59.48 (6.59) | 32 | 9.141*** | 1.95E-10 |  | 0.51 (0.40)  -0.46 (0.38) | 32 | 9.162*** | 1.84E-10 |
|  | vs. C | 65.38 (8.45)  58.45 (6.53) | 24 | 9.820*** | 7.00E-10 |  | 0.59 (0.41)  -0.69 (0.38) | 24 | 10.858*** | 9.62E-11 |
| A2 | vs. A3 | 61.12 (8.75)  61.13 (8.11) | 14 | -0.024 | .982 |  | -0.00 (0.33)  -0.05 (0.31) | 14 | 0.530 | .605 |
|  | vs. B1 | 61.30 (7.78)  57.84 (6.85) | 19 | 7.429*** | 4.94E-7 |  | 0.05 (0.35)  -0.27 (0.38) | 19 | 2.628* | .017 |
|  | vs. B2/3 | 61.42 (8.17)  57.55 (6.55) | 17 | 5.055*** | 9.76E-5 |  | 0.05 (0.37)  -0.46 (0.39) | 17 | 3.758** | .002 |
|  | vs. C | 61.16 (7.92)  56.60 (7.02) | 12 | 5.825*** | 8.15E-5 |  | 0.13 (0.40)  -0.76 (0.39) | 12 | 5.499*** | 1.37E-4 |
| A3 | vs. B1 | 61.63 (7.72)  58.13 (7.21) | 16 | 8.712*** | 1.80E-7 |  | -0.00 (0.32)  -0.23 (0.43) | 16 | 2.890* | .011 |
|  | vs. B2/3 | 61.60 (7.97)  58.07 (6.87) | 15 | 5.436*** | 6.89E-5 |  | 0.01 (0.32)  -0.45 (0.48) | 15 | 4.419*** | 4.98E-4 |
|  | vs. C | 61.51 (7.40)  57.41 (6.94) | 11 | 7.740*** | 8.93E-6 |  | 0.05 (0.35)  -0.70 (0.49) | 11 | 5.072*** | 3.60E-4 |
| B1 | vs. B2/3 | 60.20 (7.36)  59.48 (6.59) | 32 | 2.221* | .034 |  | -0.27 (0.32)  -0.46 (0.38) | 32 | 3.366** | .002 |
|  | vs. C | 60.04 (7.41)  58.45 (6.53) | 24 | 3.565** | .002 |  | -0.25 (0.32)  -0.69 (0.39) | 24 | 6.606*** | 7.83E-7 |
| B2/3 | vs. C | 59.32 (6.54)  58.45 (6.53) | 24 | 2.825** | .009 |  | -0.48 (0.39)  -0.69 (0.38) | 24 | 3.981** | .001 |

^a^ Based on a minimum criterion of 10 epochs, there were no enough subjects (n≤10) in some comparisons

* *p*<.05 ** *p*<.01 *** *p*<.001

SD=standard deviation

df=degrees of freedom

**Table S9. Results of paired sample t-tests for heart rate and skin conductance level between different EEG-vigilance stages in time block min 31-60 in the attended condition**

| attended condition | | heart rate | | | |  | skin conductance level | | | |
| --- | --- | --- | --- | --- | --- | --- | --- | --- | --- | --- |
|  |  |  | effect | | |  |  | effect | | |
| comparison | | mean (SD) | df | t | *p* |  | mean (SD) | df | t | *p* |
| 0 | vs. A1 | 67.81 (9.67)  67.26 (9.92) | 22 | 1.112 | .278 |  | 0.07 (0.41)  0.36 (0.50) | 22 | -2.860** | .009 |
|  | vs. A2^a^ | 66.18 (11.41)  65.12 (11.86) |  |  |  |  | 0.18 (0.37)  0.01 (0.47) |  |  |  |
|  | vs. A3^a^ | 72.82 (10.47)  71.35 (10.99) |  |  |  |  | 0.07 (0.47)  -0.06 (0.66) |  |  |  |
|  | vs. B1 | 67.62 (9.97)  64.00 (9.69) | 22 | 5.163*** | 3.55E-5 |  | 0.03 (0.48)  -0.18 (0.37) | 22 | 1.931 | .066 |
|  | vs. B2/3 | 68.08 (10.14)  61.26 (9.37) | 17 | 5.967*** | 1.53E-5 |  | -0.03 (0.52)  -0.42 (0.36) | 17 | 2.044 | .057 |
|  | vs. C^a^ | 69.75 (10.26)  59.67 (9.76) |  |  |  |  | 0.21 (0.48)  -0.65 (0.59) |  |  |  |
| A1 | vs. A2 | 64.18 (10.81)  63.50 (10.61) | 11 | 2.072 | .063 |  | 0.25 (0.31)  -0.02 (0.39) | 11 | 2.017 | .069 |
|  | vs. A3 | 64.74 (11.47)  63.89 (10.92) | 10 | 2.312* | .043 |  | 0.32 (0.35)  -0.03 (0.44) | 10 | 2.349* | .041 |
|  | vs. B1 | 65.20 (9.44)  61.90 (8.87) | 35 | 7.115*** | 2.72E-8 |  | 0.37 (0.44)  -0.18 (0.34) | 35 | 5.410*** | 4.63E-6 |
|  | vs. B2/3 | 65.06 (9.37)  60.27 (8.21) | 31 | 6.908*** | 9.55E-8 |  | 0.41 (0.46)  -0.37 (0.34) | 31 | 6.579*** | 2.39E-7 |
|  | vs. C | 64.41 (9.95)  57.58 (8.16) | 12 | 5.896*** | 7.30E-5 |  | 0.67 (0.54)  -0.68 (0.57) | 12 | 5.893*** | 7.33E-5 |
| A2 | vs. A3^a^ | 65.40 (11.00)  64.88 (10.74) |  |  |  |  | -0.02 (0.40)  -0.03 (0.51) |  |  |  |
|  | vs. B1 | 64.08 (11.19)  60.93 (10.18) |  |  |  |  | -0.03 (0.43)  -0.19 (0.39) |  |  |  |
|  | vs. B2/3^a^ | 65.54 (11.11)  60.91 (9.85) |  |  |  |  | 0.02 (0.40)  -0.37 (0.38) |  |  |  |
|  | vs. C^c^ | 72.63 ()  59.68 () |  |  |  |  | 0.81 ()  -0.83 () |  |  |  |
| A3 | vs. B1^a^ | 64.81 (11.52)  62.55 (12.04) |  |  |  |  | -0.04 (0.49)  -0.18 (0.50) |  |  |  |
|  | vs. B2/3^a^ | 61.65 (10.77)  58.53 (9.75) |  |  |  |  | 0.00 (0.48)  -0.41 (0.37) |  |  |  |
|  | vs. C^a^ | 60.19 (11.59)  54.66 (4.38) |  |  |  |  | 0.36 (0.54)  -0.96 (0.39) |  |  |  |
| B1 | vs. B2/3 | 61.30 (8.80)  59.96 (8.32) | 30 | 2.596* | .014 |  | -0.23 (0.37)  -0.39 (0.32) | 30 | 2.325* | .027 |
|  | vs. C | 60.45 (8.79)  57.58 (8.16) | 12 | 3.013* | .011 |  | -0.24 (0.35)  -0.68 (0.57) | 12 | 2.640* | .022 |
| B2/3 | vs. C | 57.77 (7.76)  57.58 (8.16) | 12 | 0.347 | .735 |  | -0.46 (0.34)  -0.68 (0.57) | 12 | 2.194* | .049 |

^a^ Based on a minimum criterion of 10 epochs, there were no enough subjects (n≤10) in some comparisons

^c^ These comparisons contained only one subject

* *p*<.05 ** *p*<.01 *** *p*<.001

SD=standard deviation

df=degrees of freedom

**Table S10. Results of paired sample t-tests for heart rate and skin conductance level between different EEG-vigilance stages in time block min 61-90 in the ignored condition**

| ignored condition | | heart rate | | | |  | skin conductance level | | | |
| --- | --- | --- | --- | --- | --- | --- | --- | --- | --- | --- |
|  |  |  | effect | | |  |  | effect | | |
| comparison | | mean (SD) | df | t | *p* |  | mean (SD) | df | t | *p* |
| 0 | vs. A1 | 61.58 (8.34)  62.40 (7.19) | 20 | -1.573 | .131 |  | 0.30 (0.66)  0.43 (0.43) | 20 | -1.025 | .317 |
|  | vs. A2 | 55.42 (5.91)  57.00 (5.84) |  |  |  |  | -0.01 (0.32)  0.10 (0.39) |  |  |  |
|  | vs. A3^a^ | 55.65 (7.13)  56.20 (5.53) |  |  |  |  | 0.18 (0.59)  -0.05 (0.29) |  |  |  |
|  | vs. B1 | 61.70 (8.25)  59.29 (6.92) | 24 | 3.710** | .001 |  | 0.24 (0.62)  -0.19 (0.33) | 24 | 3.369** | .003 |
|  | vs. B2/3 | 61.28 (8.70)  57.345 (6.29) | 17 | 3.803** | .001 |  | 0.32 (0.72)  -0.50 (0.38) | 17 | 4.264** | .001 |
|  | vs. C^a^ | 65.21 (8.91)  58.54 (5.47) |  |  |  |  | 0.76 (0.84)  -0.73 (0.34) |  |  |  |
| A1 | vs. A2 | 60.81 (7.55)  59.98 (7.15) | 16 | 2.542* | .022 |  | 0.39 (0.39)  0.09 (0.33) | 16 | 3.449** | .003 |
|  | vs. A3 | 60.23 (7.56)  58.37 (6.53) | 12 | 2.511* | .027 |  | 0.53 (0.42)  -0.07 (0.24) | 12 | 5.626*** | 1.11E-4 |
|  | vs. B1 | 64.17 (7.79)  59.52 (6.79) | 29 | 7.916*** | 9.90E-9 |  | 0.49 (0.48)  -0.28 (0.31) | 29 | 8.228*** | 4.52E-9 |
|  | vs. B2/3 | 64.37 (8.03)  58.59 (6.52) | 26 | 8.385*** | 7.25E-9 |  | 0.53 (0.49)  -0.48 (0.36) | 26 | 8.173*** | 1.18E-8 |
|  | vs. C | 67.87 (6.86)  59.73 (5.63) | 12 | 9.855*** | 4.19E-7 |  | 0.85 (0.51)  -0.63 (0.44) | 12 | 7.896*** | 4.30E-6 |
| A2 | vs. A3 | 58.85 (7.23)  58.30 (6.82) | 11 | 1.120 | .287 |  | 0.08 (0.38)  -0.08 (0.25) | 11 | 1.660 | .125 |
|  | vs. B1 | 60.10 (7.09)  56.60 (6.64) | 16 | 6.098*** | 1.54E-5 |  | 0.10 (0.32)  -0.32 (0.34) | 16 | 3.588** | .002 |
|  | vs. B2/3 | 60.77 (7.52)  57.05 (7.07) | 15 | 4.155** | .001 |  | 0.10 (0.34)  -0.38 (0.32) | 15 | 4.076** | .001 |
|  | vs. C^a^ | 63.53 (5.84)  57.12 (6.02) |  |  |  |  | 0.15 (0.41)  -0.52 (0.53) |  |  |  |
| A3 | vs. B1 | 58.44 (6.82)  55.48 (6.41) | 11 | 5.226*** | 2.83E-4 |  | -0.06 (0.25)  -0.29 (0.38) | 11 | 2.646* | .023 |
|  | vs. B2/3 | 58.34 (6.82)  55.22 (5.89) | 11 | 3.662** | .004 |  | -0.06 (0.25)  -0.44 (0.27) | 11 | 2.952* | .013 |
|  | vs. C^a^ | 62.37 (6.21)  58.03 (5.74) |  |  |  |  | 0.04 (0.29)  -0.66 (0.28) |  |  |  |
| B1 | vs. B2/3 | 59.53 (6.75)  58.60 (6.47) | 27 | 2.744* | .011 |  | -0.29 (0.35)  -0.52 (0.38) | 27 | 2.864** | .008 |
|  | vs. C | 61.15 (5.37)  59.73 (5.63) | 12 | 3.506** | .004 |  | -0.13 (0.25)  -0.63 (0.44) | 12 | 3.433** | .005 |
| B2/3 | vs. C | 59.83 (5.55)  59.73 (5.63) | 12 | 0.411 | .688 |  | -0.52 (0.33)  -0.63 (0.44) | 12 | 1.569 | .143 |

^a^ Based on a minimum criterion of 10 epochs, there were no enough subjects (n≤10) in some comparisons

* *p*<.05 ** *p*<.01 *** *p*<.001

SD=standard deviation

df=degrees of freedom

**Table S11. Results of paired sample t-tests for heart rate and skin conductance level between different EEG-vigilance stages in time block min 61-90 in the attended condition**

| attended condition | | heart rate | | | |  | skin conductance level | | | |
| --- | --- | --- | --- | --- | --- | --- | --- | --- | --- | --- |
|  |  |  | effect | | |  |  | effect | | |
| comparison | | mean (SD) | df | t | *p* |  | mean (SD) | df | t | *p* |
| 0 | vs. A1 | 65.82 (10.21)  66.00 (9.65) | 24 | -0.283 | .780 |  | 0.14 (0.62)  0.30 (0.44) | 24 | -1.183 | .082 |
|  | vs. A2^a^ | 63.96 (11.17)  64.22 (10.83) |  |  |  |  | 0.04 (0.58)  -0.06 (0.24) |  |  |  |
|  | vs. A3^a^ | 63.43 (12.14)  62.93 (12.42) |  |  |  |  | 0.00 (0.55)  -0.23 (0.34) |  |  |  |
|  | vs. B1 | 65.98 (9.99)  62.85 (10.13) | 24 | 4.162*** | 3.50E-4 |  | 0.17 (0.61)  -0.22 (0.35) | 24 | 2.713* | .012 |
|  | vs. B2/3 | 63.71 (10.26)  59.10 (9.58) | 17 | 4.019** | .001 |  | 0.11 (0.70)  -0.37 (0.43) | 17 | 2.114 | .050 |
|  | vs. C^a^ | 63.04 (9.59)  55.33 (6.23) |  |  |  |  | 0.37 (0.92)  -0.48 (0.76) |  |  |  |
| A1 | vs. A2 | 63.66 (9.56)  62.24 (9.41) | 11 | 3.431** | .006 |  | 0.19 (0.25)  0.01 (0.20) | 11 | 1.657 | .126 |
|  | vs. A3 | 62.42 (10.03)  60.66 (10.35) | 13 | 4.896*** | 2.92E-4 |  | 0.19 (0.23)  -0.19 (0.30) | 13 | 2.875* | .013 |
|  | vs. B1 | 65.13 (9.36)  61.45 (9.61) | 33 | 7.518*** | 1.20E-8 |  | 0.28 (0.39)  -0.20 (0.39) | 33 | 4.560*** | 6.70E-5 |
|  | vs. B2/3 | 63.09 (8.73)  58.62 (8.56) | 28 | 7.076*** | 1.07E-7 |  | 0.31 (0.42)  -0.32 (0.44) | 28 | 4.563*** | 9.15E-5 |
|  | vs. C | 61.90 (7.69)  54.79 (5.29) | 14 | 6.539*** | 1.31E-5 |  | 0.49 (0.49)  -0.83 (0.71) | 14 | 5.655*** | 5.92E-5 |
| A2 | vs. A3 | 61.70 (9.67)  61.30 (9.41) | 10 | 1.447 | .179 |  | 0.03 (0.20)  -0.11 (0.28) | 10 | 1.873 | .091 |
|  | vs. B1 | 62.60 (9.88)  60.22 (9.86) |  |  |  |  | -0.02 (0.21)  -0.10 (0.48) |  |  |  |
|  | vs. B2/3 | 60.76 (9.68)  58.40 (10.11) |  |  |  |  | -0.02 (0.21)  -0.27 (0.44) |  |  |  |
|  | vs. C^a^ | 59.02 (5.81)  55.20 (3.03) |  |  |  |  | 0.04 (0.22)  -1.03 (0.33) |  |  |  |
| A3 | vs. B1 | 60.81 (10.93)  58.87 (10.98) | 11 | 3.377** | .006 |  | -0.22 (0.31)  -0.15 (0.46) | 11 | -0.799 | .441 |
|  | vs. B2/3 | 59.28 (10.57)  57.26 (10.46) | 11 | 2.684* | .023 |  | -0.20 (0.32)  -0.25 (0.42) | 11 | 0.373 | .716 |
|  | vs. C^a^ | 55.62 (6.14)  52.59 (4.73) |  |  |  |  | -0.36 (0.32)  -1.06 (0.36) |  |  |  |
| B1 | vs. B2/3 | 58.89 (8.58)  52.59 (4.73) | 25 | 2.244* | .034 |  | -0.20 (0.39)  -0.36 (0.45) | 25 | 2.315* | .029 |
|  | vs. C | 56.80 (6.87)  54.79 (5.29) | 14 | 2.932* | .011 |  | -0.28 (0.36)  -0.83 (0.71) | 14 | 2.843* | .013 |
| B2/3 | vs. C | 55.79 (6.35)  54.79 (5.29) | 14 | 1.785 | .096 |  | -0.51 (0.42)  -0.83 (0.71) | 14 | 2.037 | .061 |

^a^ Based on a minimum criterion of 10 epochs, there were no enough subjects (n≤10) in some comparisons

* *p*<.05 ** *p*<.01 *** *p*<.001

SD=standard deviation

df=degrees of freedom

**Table S12. Results of paired sample t-tests for heart rate and skin conductance level between different EEG-vigilance stages in time block min 91-120 in the ignored condition**

| ignored condition | | heart rate | | | |  | skin conductance level | | | |
| --- | --- | --- | --- | --- | --- | --- | --- | --- | --- | --- |
|  |  |  | effect | | |  |  | effect | | |
| comparison | | mean (SD) | df | t | *p* |  | mean (SD) | df | t | *p* |
| 0 | vs. A1 | 63.37 (7.96)  63.96 (6.98) | 22 | -1.347 | .192 |  | 0.07 (0.38)  0.31 (0.42) | 22 | -2.974** | .007 |
|  | vs. A2 | 61.87 (10.20)  61.54 (7.85) | 10 | 0.333 | .746 |  | -0.11 (0.49)  0.09 (0.32) | 10 | -1.108 | .294 |
|  | vs. A3 | 60.97 (8.72)  59.20 (7.04) |  |  |  |  | 0.06 (0.67)  -0.18 (0.38) |  |  |  |
|  | vs. B1 | 62.55 (8.37)  59.73 (7.41) | 27 | 4.011*** | 4.30E-4 |  | -0.03 (0.46)  -0.26 (0.39) | 27 | 3.100** | .004 |
|  | vs. B2/3 | 62.51 (8.99)  57.46 (6.66) | 18 | 5.399*** | 3.95E-5 |  | -0.02 (0.53)  -0.55 (0.34) | 18 | 4.132** | .001 |
|  | vs. C^a^ | 64.28 (10.45)  56.98 (7.60) |  |  |  |  | 0.15 (0.57)  -0.71 (0.20) |  |  |  |
| A1 | vs. A2 | 62.32 (7.51)  61.51 (7.63) | 11 | 3.109* | .010 |  | 0.25 (0.31)  0.08 (0.37) | 11 | 1.923 | .081 |
|  | vs. A3 | 62.89 (8.39)  60.15 (7.99) | 11 | 2.606* | .024 |  | 0.47 (0.53)  -0.10 (0.31) | 11 | 2.923* | .014 |
|  | vs. B1 | 65.46 (8.97)  60.91 (7.55) | 28 | 6.203*** | 1.06E-6 |  | 0.33 (0.40)  -0.19 (0.39) | 28 | 5.019*** | 2.63E-5 |
|  | vs. B2/3 | 65.81 (9.51)  59.34 (7.60) | 23 | 7.565*** | 1.10E-7 |  | 0.41 (0.41)  -0.49 (0.37) | 23 | 6.864*** | 5.34E-7 |
|  | vs. C | 66.65 (8.88)  59.17 (7.78) | 10 | 10.869*** | 7.37E-7 |  | 0.67 (0.46)  -0.74 (0.26) | 10 | 9.120*** | 3.67E-6 |
| A2 | vs. A3^a^ | 59.25 (6.99)  58.75 (6.66) |  |  |  |  | 0.05 (0.39)  -0.07 (0.38) |  |  |  |
|  | vs. B1 | 62.14 (8.19)  59.74 (8.33) | 14 | 4.056** | .001 |  | 0.09 (0.32)  -0.11 (0.53) | 14 | 1.186 | .255 |
|  | vs. B2/3 | 63.63 (9.04)  61.24 (9.78) | 13 | 2.511* | .026 |  | -0.00 (0.37)  -0.35 (0.45) | 13 | 1.964 | .071 |
|  | vs. C | 62.55 (9.37)  56.67 (8.21) |  |  |  |  | 0.31 (0.46)  -0.77 (0.23) |  |  |  |
| A3 | vs. B1 | 59.92 (8.14)  56.96 (7.45) | 11 | 3.686** | .004 |  | -0.17 (0.34)  -0.13 (0.57) | 11 | -0.280 | .784 |
|  | vs. B2/3 | 59.08 (7.93)  55.86 (6.96) | 11 | 2.576* | .026 |  | -0.17 (0.34)  -0.47 (0.47) | 11 | 2.218* | .049 |
|  | vs. C^a^ | 61.17 (8.55)  57.38 (8.21) |  |  |  |  | -0.11 (0.34)  -0.82 (0.28) |  |  |  |
| B1 | vs. B2/3 | 60.37 (8.33)  59.44 (8.12) | 25 | 2.797* | .010 |  | -0.28 (0.45)  -0.50 (0.40) | 25 | 3.084** | .005 |
|  | vs. C | 59.42 (6.95)  59.17 (7.78) | 10 | 0.334 | .745 |  | -0.21 (0.31)  -0.74 (0.26) | 10 | 4.717** | .001 |
| B2/3 | vs. C | 58.06 (6.89)  59.17 (7.78) | 10 | -1.499 | .165 |  | -0.55 (0.20)  -0.74 (0.26) | 10 | 3.884** | .003 |

^a^ Based on a minimum criterion of 10 epochs, there were no enough subjects (n≤10) in some comparisons

* *p*<.05 ** *p*<.01 *** *p*<.001

SD=standard deviation

df=degrees of freedom

**Table S13. Results of paired sample t-tests for heart rate and skin conductance level between different EEG-vigilance stages in time block min 91-120 in the attended condition**

| attended condition | | heart rate | | | |  | skin conductance level | | | |
| --- | --- | --- | --- | --- | --- | --- | --- | --- | --- | --- |
|  |  |  | effect | | |  |  | effect | | |
| comparison | | mean (SD) | df | t | *p* |  | mean (SD) | df | t | *p* |
| 0 | vs. A1 | 65.66 (8.41)  66.56 (8.87) | 24 | -1.467 | .155 |  | 0.17 (0.56)  0.19 (0.36) | 24 | -0.242 | .811 |
|  | vs. A2^a^ | 67.23 (8.72)  67.42 (9.40) |  |  |  |  | 0.12 (.036)  -0.00 (0.29) |  |  |  |
|  | vs. A3^a^ | 67.31 (9.74)  67.12 (10.08) |  |  |  |  | -0.06 (0.24)  -0.22 (0.32) |  |  |  |
|  | vs. B1 | 64.82 (8.69)  63.44 (8.65) | 26 | 2.542* | .017 |  | 0.14 (0.55)  -0.11 (0.38) | 26 | 2.455* | .021 |
|  | vs. B2/3 | 64.48 (8.38)  61.00 (7.97) | 13 | 2.764* | .016 |  | 0.31 (0.70)  -0.18 (0.36) | 13 | 2.500* | .027 |
|  | vs. C^a^ | 63.06 (4.27)  55.01 (2.16) |  |  |  |  | 1.33 (0.95)  -0.61 (0.05) |  |  |  |
| A1 | vs. A2 | 64.17 (10.40)  63.30 (10.73) | 12 | 3.566** | .004 |  | 0.17 (0.18)  -0.05 (0.27) | 12 | 2.321* | .039 |
|  | vs. A3 | 63.61 (10.96)  62.23 (10.81) | 10 | 4.324** | .002 |  | 0.15 (0.16)  -0.25 (0.34) | 10 | 3.099* | .011 |
|  | vs. B1 | 65.08 (8.98)  62.15 (8.60) | 33 | 6.101*** | 7.17E-7 |  | 0.21 (0.32)  -0.16 (0.36) | 33 | 4.212*** | 1.83E-4 |
|  | vs. B2/3 | 64.18 (9.11)  59.67 (8.32) | 23 | 5.592*** | 1.09E-5 |  | 0.26 (0.37)  -0.30 (0.39) | 23 | 4.185*** | 3.55E-4 |
|  | vs. C^a^ | 61.57 (7.61)  54.70 (4.18) |  |  |  |  | 0.46 (0.50)  -0.78 (0.50) |  |  |  |
| A2 | vs. A3 | 62.97 (11.32)  62.23 (10.81) | 10 | 1.771 | .107 |  | -0.10 (0.25)  -0.25 (0.34) | 10 | 1.738 | .113 |
|  | vs. B1 | 63.18 (10.42)  61.10 (9.60) | 10 | 2.689* | .023 |  | -0.07 (0.29)  -0.10 (0.46) | 10 | 0.241 | .814 |
|  | vs. B2/3 | 61.56 (11.65)  59.59 (11.37) |  |  |  |  | 0.01 (0.27)  -0.31 (0.33) |  |  |  |
|  | vs. C^a^ | 57.96 (8.75)  53.67 (5.25) |  |  |  |  | -0.04 (0.07)  -0.64 (0.26) |  |  |  |
| A3 | vs. B1^a^ | 62.09 (10.78)  60.76 (10.29) |  |  |  |  | -0.26 (0.38)  -0.14 (0.51) |  |  |  |
|  | vs. B2/3^a^ | 60.14 (11.92)  59.29 (12.33) |  |  |  |  | -0.27 (0.35)  -0.31 (0.38) |  |  |  |
|  | vs. C^a^ | 58.00 (9.51)  53.67 (5.25) |  |  |  |  | -0.11 (0.35)  -0.64 (0.26) |  |  |  |
| B1 | vs. B2/3 | 59.92 (7.71)  58.88 (7.54) | 22 | 2.464* | .022 |  | -0.17 (0.40)  -0.30 (0.39) | 22 | 1.621 | .119 |
|  | vs. C^a^ | 56.94 (5.50)  54.70 (4.18) |  |  |  |  | -0.22 (0.28)  -0.78 (0.50) |  |  |  |
| B2/3 | vs. C^a^ | 55.70 (4.55)  54.70 (4.18) |  |  |  |  | -0.47 (0.31)  -0.78 (0.50) |  |  |  |

^a^ Based on a minimum criterion of 10 epochs, there were no enough subjects (n≤10) in some comparisons

* *p*<.05 ** *p*<.01 *** *p*<.001

SD=standard deviation

df=degrees of freedom
